# Syphilis self-testing and implications for syphilis control and prevention

**DOI:** 10.1128/jcm.00982-25

**Published:** 2025-10-21

**Authors:** Ayel Luis R. Batac, Michael Marks, Joseph D. Tucker, Rosanna Ŵ. Peeling

**Affiliations:** 1 Faculty of Public Health and Policy, London School of Hygiene & Tropical Medicine 4906, London, United Kingdom; 2 Institute for Global Health and Infectious Diseases, School of Medicine, University of North Carolina at Chapel Hill, Chapel Hill, North Carolina, USA; 3 Department of Clinical Research, Faculty of Infectious and Tropical Diseases, London School of Hygiene & Tropical Medicine 4906, London, United Kingdom; 4 Hospital for Tropical Diseases, University College London Hospitals NHS Foundation Trust 8964, London, United Kingdom; 5 Division of Infection and Immunity, Faculty of Medical Sciences, University College London 4919, London, United Kingdom; 6 International Diagnostics Centre, London School of Hygiene & Tropical Medicine 4906, London, United Kingdom; 7 Division of Infectious Diseases, Department of Medicine, School of Medicine, University of North Carolina at Chapel Hill 2331, Chapel Hill, North Carolina, USA; 8 Department of Medical Microbiology and Infectious Diseases, Rady Faculty of Health Sciences, Max Rady College of Medicine, University of Manitoba 8664, Winnipeg, Manitoba, Canada; Marquette University, Milwaukee, Wisconsin, USA

**Keywords:** syphilis, *Treponema pallidum*, diagnostics, rapid syphilis test, self-test, over-the-counter syphilis test

## Abstract

The incidence of syphilis has risen dramatically in many regions despite the availability of affordable facility-based testing and curative treatment. The recent approval of over-the-counter syphilis self-tests (SSTs) represents an important advance for expanding diagnostic access and disease control and prevention. Evidence demonstrates that SSTs are accurate, usable, and acceptable. However, as with HIV and COVID-19 self-testing, implementation challenges remain, including ensuring equitable access, supporting vulnerable groups, securing linkage to care, and maintaining quality assurance.

## INTRODUCTION

Syphilis is resurging globally, with the United States reporting its highest case counts in decades (1) and similar increases observed across multiple regions (2). These trends reflect missed opportunities for prevention and diagnosis, with profound consequences, including congenital infections (3). Although curable with antibiotics, delayed treatment can lead to severe morbidity and mortality, and early symptoms are often absent or non-specific, hindering timely recognition and care (4).

Despite advances in diagnostics, facility-based syphilis testing remains inaccessible for many, including men who have sex with men (MSM), sex workers, and pregnant women (5–7). Barriers such as stigma, inconvenient clinic hours, and long travel distances limit uptake. To address these gaps, syphilis self-testing (SST) has emerged. In a *Journal of Clinical Microbiology* article by K. Clark et al. (63:e00244-25, 2025, https://doi.org/10.1128/jcm.00244-25), the authors demonstrated that syphilis self-testing (SST) is accurate and feasible for over-the-counter use. SST enables individuals to collect their own specimens, perform the test, and interpret results independently (8).

The approval of the first SST represents a turning point in syphilis control. Yet questions remain: Can SST meaningfully expand access to diagnostics? How can scale-up ensure equity, safety, and quality? How can we embed SST within existing HIV self-testing (HIVST) infrastructure and programs, drawing on lessons already learned? In this commentary, we describe the limitations of facility-based syphilis testing and critically examine the promise and challenges of SST.

## LIMITATIONS OF FACILITY-BASED SYPHILIS TESTING

Facility-based testing remains the cornerstone of syphilis diagnosis and surveillance in most countries. Yet, despite its clinical accuracy and established infrastructure, access remains limited for many high-risk groups. Stigma, discrimination, and breaches of confidentiality deter MSM, sex workers, and pregnant women from attending clinics (5). Geographic distance, restrictive clinic hours, and financial costs further discourage attendance. In addition, multi-step pathways that involve separate visits for assessment, testing, and treatment increase the likelihood of loss to follow-up.

Health system limitations also contribute to delays in diagnosis. Shortages of trained personnel, reagent stockouts, overcrowded facilities, and reliance on reference laboratories for confirmatory testing all impede efficient service delivery (6). In some settings, only part of the recommended diagnostic algorithm is performed on-site, with specimens sent to reference laboratories for confirmation, introducing further delays in initiating treatment. These barriers underscore the need for complementary models like SST, which can bypass many obstacles by providing immediate and private results, provided that linkage to care and integration with surveillance are ensured.

**Fig 1:**
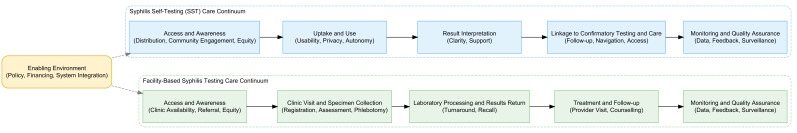
Comparative care continuum for SST and facility-based testing, illustrating sequential stages from access and awareness to monitoring and quality assurance.

The differences between facility-based and self-testing pathways are summarized in Fig. 1.

## SYPHILIS SELF-TESTING

Most SSTs detect treponemal antibodies to *Treponema pallidum*, which typically remain positive for life, indicating past or present infection (8). Lipoidal tests such as rapid plasma reagin and Venereal Disease Research Laboratory assays measure antibody–lipid complexes that decline after treatment and can monitor disease activity (8, 9). These require serum and laboratory infrastructure, making them unsuitable for self-testing. Currently, all SSTs are treponemal-based, including some dual HIV/syphilis tests.

The First To Know Syphilis Test (NOWDiagnostics, Inc., Springdale, Arkansas, United States) is a single-use, treponemal antibody rapid self-test evaluated for over-the-counter use (8). Requiring only a finger-prick blood sample, it provides results within minutes and was designed for ease of use across literacy levels. Unlike many rapid tests, it does not require liquid handling because all reagents are dried inside the cassette and automatically rehydrate on contact with the specimen. This feature simplifies both test shipment and user performance, making the device particularly well-suited for decentralized use.

The World Health Organization (WHO) recognizes self-testing as a core self-care intervention and provides guidance for validation and use (10). The United States Food and Drug Administration recently authorized the First To Know Test after data showed high sensitivity and specificity consistent with WHO standards (≥85% sensitivity, ≥95% specificity compared to laboratory-based treponemal assays). In Europe, the Syphilis Home Test (Patris Health, Ljubljana, Slovenia) carries CE marking, while dual HIV/syphilis rapid tests have been prequalified by WHO and licensed in several regions for point-of-care use. These milestones reflect growing recognition of SST’s public health value.

Implementation, however, remains uneven. The United States leads on regulatory approval, while China has pioneered practical delivery, distributing bundled HIVST–SST at scale (11, 12). Trials in China highlight community-based distribution, integration with HIV programs, and user preferences. In the Netherlands, regulatory uncertainty and limited demand outside MSM slowed integration (13). In Zimbabwe, uptake was high among MSM but hindered by stigma, criminalization, weak supply chains, and absent policy frameworks (14, 15).

Scaling up SST will require strategies tailored to local contexts. In high-income countries, this means clearer regulatory pathways and initiatives to generate demand. In lower-income settings, priorities include investment in supply chains, supportive national policies, and funding models that make tests affordable or available free of charge. Across all settings, integration into health systems, with strong linkage to care, effective surveillance, and efforts to reduce stigma, will be critical to maximizing public health impact.

## EVIDENCE AND OPPORTUNITIES FOR SST

Evidence from multiple settings demonstrates that SST increases uptake, particularly among first-time testers. In a three-arm randomized controlled trial of 451 MSM across 124 cities in China, participants were allocated to standard of care, standard SST, or SST with financial incentives. Confirmed syphilis testing within 6 months was significantly higher in both SST arms (63.4% [90/142] and 65.7% [90/137]) compared with standard of care (14.7% [20/136]) (12). The risk differences were 48.7% and 51.0%, respectively (both *P* < 0.001). Nearly 80% of self-testers were first-time syphilis testers, underscoring its potential to reach individuals not engaged by facility-based services (12). The intervention was also more cost-efficient: the estimated cost per person tested was 26.55–28.09 USD in the SST arms compared with 66.19 USD under standard care, largely due to reduced personnel and facility overheads.

Survey and observational studies corroborate these findings. A nationwide survey of 699 MSM in China reported that 24.9% (174/699) had ever self-tested for syphilis; more than half were first-time testers, and most paired SST with HIVST (11). Among those with a reactive result, 83.3% (30/36) sought confirmatory care, typically within 1 month. Among female sex workers in eight Chinese cities, 5.9% (76/1,287) reported ever using SST, with 57.9% (44/76) being first-time testers (7). Care-seeking was high: 86.9% (20/23) of women with reactive or uncertain results obtained follow-up, most within 2 weeks. Predictors of uptake included higher-risk behaviors such as recent anal sex (aOR 2.6, 95% CI 1.5–4.3), substance use during sex (aOR 3.8, 95% CI 2.3–6.4), and prior sexually transmitted infections (STI) testing (aOR 3.4, 95% CI 1.9–6.0). Additional observational work supports feasibility: Wu et al. found that 99% (1,141/1,150) of MSM returned valid results after social media-based secondary distribution of HIVST–SST kits (16). Zhong et al. reported 90% (178/198) of MSM completed HIVST–SST kits within 4 weeks of purchase (17). Beyond uptake, digital tools may enhance feasibility and user experience. In the United States, Balán et al. evaluated the SMARTtest app integrating dual HIVST–SST with support features; all 59 MSM in the study successfully used it, and mini-pilot participants reported it was easy to use and comprehensive, indicating high acceptability (18).

These data highlight both the feasibility and accessibility of SST. Beyond formal evaluations, kits are available through online and community channels for 1–5 USD, a price point likely to facilitate uptake among underserved groups (11, 16). The WHO’s 2024 guidance reflects this evidence, recommending SST as an additional approach for high-risk and underserved populations, with emphasis on integration into confirmatory pathways, provision of clear user instructions, and adaptation to local epidemiology (19). Table 1 summarizes these recommendations. In high-incidence settings, widespread distribution may accelerate case detection and reduce transmission, while in lower-incidence contexts, innovative funding models and targeted distribution to key populations and first-time testers may maximize cost-effectiveness.

**TABLE 1:** WHO 2024 recommendations for SST (adapted from [19, 20])

Recommendation area	Summary of WHO 2024 guidance
Role of SST	SST is recommended as an *additional* syphilis testing approach, especially for underserved and high-risk populations, complementing facility-based testing.
Product quality	Only use quality-assured, validated SST products that meet or exceed WHO performance thresholds for rapid treponemal tests.
Integration with services	SST should be linked to confirmatory testing, treatment, and partner services via clear referral pathways.
User instructions	Provide simple, clear, language-appropriate instructions, with visual aids where possible, to support correct use and result interpretation.
Adaptation to local context	Tailor SST delivery to local epidemiology, health system capacity, cultural factors, and user needs.
Distribution models	Use flexible channels such as community distribution, pharmacies, online platforms, and integration with HIVST where feasible.
Monitoring and evaluation	Include SST in surveillance systems and program monitoring to track uptake, linkage to care, and outcomes.

## LIMITATIONS OF SST

Despite its promise, SST faces technical, social, and equity challenges that require careful attention. Treponemal antibody assays, which underpin all current SSTs, cannot distinguish between active infection and previously treated infection (8, 21, 22). This limitation also makes SST less useful in populations at high risk of reinfection, such as MSM and sex workers. Package inserts generally note that a reactive result may indicate past or present infection and recommend confirmatory testing, but clearer warnings are needed to reduce confusion, anxiety, or inappropriate retreatment. Performance in pregnancy also warrants caution: to date, the only published study evaluating SST in this population, by Clark et al., reported a sensitivity of 75.0% (95% CI, 40.9%–92.9%) among pregnant women compared with 94.9% (95% CI, 88.5%–98.3%) in sexually active adults (8). Although based on small numbers, this finding underscores the need for larger studies in antenatal populations; until then, SST should not be promoted as a primary diagnostic tool in pregnancy without confirmatory testing (22, 23).

Social and equity considerations are equally important. While adverse biological outcomes have not been reported, some participants in China and the United States described pressure from partners or peers to self-test (7, 11, 16, 18). Although uncommon, such coercion undermines voluntariness and highlights the need for safeguards that promote autonomy. Access pathways also risk reinforcing disparities: reliance on online sales or pharmacies may exclude those without internet access, stable delivery addresses, or disposable income. To ensure equitable reach, SST should be integrated into public-sector and community-based channels, with subsidized or free provision prioritized for high-risk and underserved populations.

## LEARNING FROM HIVST

The global expansion of HIVST offers important lessons for the scale-up of SST. By mid-2024, 107 countries had adopted HIVST policies, with 71 implementing them routinely (20). Across diverse contexts, HIVST has consistently increased testing uptake, frequency, and coverage, particularly among populations underserved by facility-based services such as MSM, sex workers, transgender individuals, and adolescents (20, 24, 25). For many, HIVST represented a first opportunity to know their status, illustrating how self-care diagnostics can reach individuals missed by clinics. These experiences demonstrate the feasibility of normalizing self-testing at scale while highlighting challenges that SST must anticipate.

A central lesson is the persistent gap between self-testing and linkage to confirmatory services. While HIVST increased knowledge of HIV status, delays in confirmatory testing and treatment were common, particularly in settings with fragmented systems (25). Effective linkage strategies included SMS reminders, telephone hotlines, web-based platforms, mobile applications, and support from peers or community health workers, all of which promoted timely care and provided reassurance. Usability supports also proved critical: HIVST studies showed that simple written and pictorial instructions improved accuracy, while additional aids such as apps, instructional videos, and hotlines further reduced misinterpretation and improved confidence (25, 26). The WHO has further emphasized that usability barriers disproportionately affect persons with disabilities, older adults, and other marginalized groups, recommending accessible features such as clear instructions, intuitive visuals, and user-centric components (22). Together, these findings highlight that the value of self-testing depends not only on diagnostic performance but also on the presence of user-centered support systems. Table 2 summarizes comparative insights across HIVST, SST, and dual HIVST–SST platforms, highlighting shared strengths as well as distinct challenges and illustrating how lessons from HIVST can inform scale-up of SST.

**TABLE 2:** Similarities and differences between HIVST, SST, and dual HIVST–SST in terms of advantages, limitations, and implementation status^*a*^

Feature	HIVST	SST	Dual HIV–SST
Availability and status	Widely available >100 countries have policies supporting HIVST Many have integrated into routine practice	Recently introduced FDA-authorized over-the-counter test in the United States Pilot programs and research in China, Zimbabwe, and elsewhere	Available in some countries for clinic or community use WHO-prequalified dual rapid diagnostic tests for point-of-care Limited self-use approvals Piloted at scale in China
Diagnostic target	Detects HIV antibodies (third- and fourth-generation tests)	Most are treponemal antibody tests Cannot distinguish active from past infection	Detects HIV antibodies and treponemal antibodies in the same device Same SST limitation on distinguishing past vs active syphilis
Advantages	High acceptability and uptake Reduces stigma Empowers users Enables frequent and partner testing Strong evidence base Digital and peer support models	High acceptability and usability in studies Potential to close testing gaps among key populations Can be bundled with HIVST Over-the-counter and community models emerging	Offers convenience of single sample for two infections Potential cost and distribution efficiencies Aligns with integrated testing strategies Feasible for antenatal care and key populations
Limitations	Linkage to confirmatory care can be suboptimal Window period limits early detection Potential for user error Variable regulation and quality assurance	Cannot distinguish between past and current infection Limited sensitivity in some groups (e.g., pregnant women) Risk of user error and misinterpretation Limited real-world data Linkage to care pathways not yet widely established	Inherits limitations from both HIVST and SST Slightly more complex interpretation Higher cost per unit Regulatory approvals more complex Limited self-use data
Equity considerations	Programs address access for key populations Subsidized or free in many settings	Equity gaps possible if only over-the-counter/online Need for subsidized and accessible distribution channels	Potentially increases efficiency of outreach to underserved populations Equity risks if more expensive and not subsidized
Integration with health systems	Increasingly integrated with digital health tools, community delivery, and reporting systems	Integration with HIVST in some countries (dual tests) Not yet routine in most health systems	Facilitates integrated service delivery Potential to streamline antenatal care and key population testing Requires coordination of confirmatory pathways for both infections
Evidence base	Extensive trials, implementation studies, and systematic reviews support effectiveness and safety	Emerging evidence from United States, China, Zimbabwe, and systematic reviews Long-term effectiveness still under study	Early programmatic and research evidence from China, antenatal care programs, and WHO-prequalified rapid diagnostic test evaluations Limited formal self-use studies

^*a*^ FDA, Food and Drug Administration.

HIVST scale-up also underscores broader structural issues relevant to SST. Procurement and financing were often slowed by fragmented donor support, weak supply chains, and lack of integration into national budgets (27). Embedding HIVST into procurement systems and universal health coverage frameworks proved essential for sustainability, and similar planning will be required for SST. Integration of dual HIV/syphilis tests, already implemented at scale in China (7, 11, 12), demonstrates the potential for bundled approaches but requires harmonized regulatory approvals, aligned counseling materials, and coordinated supply systems. Collectively, HIVST shows that self-testing can expand access, reduce stigma, and empower individuals when supported by comprehensive systems of linkage, usability support, and sustainable financing. Applying these lessons to syphilis provides a pragmatic roadmap for translating diagnostic innovation into meaningful public health impact.

## IMPLEMENTATION CHALLENGES, RESEARCH PRIORITIES, AND THE FUTURE OF SST

The future of SST depends on demonstrating tangible public health benefits: earlier diagnosis, timely treatment, and reduced transmission. Current evidence comes largely from pilot studies in specific populations, particularly MSM in China, with smaller studies in other contexts (7, 11, 12). Research must extend to pregnant women, sex workers, adolescents, and people living with HIV, where delayed diagnosis carries serious consequences, such as vertical transmission in pregnancy (23). Dual or bundled HIVST–SST platforms, already piloted at scale in China, also require comparative evaluation to determine their cost-effectiveness and acceptability relative to single-disease testing.

Sustaining linkage to care remains a central challenge. While encouraging rates have been observed in pilot studies, delays are common (11). Building on HIVST experience, future SST programs should adapt proven strategies such as digital support, automated reminders, and peer- or partner-led navigation, but formal evaluation is needed to confirm their effectiveness. The WHO increasingly promotes self-testing as part of its broader self-care agenda (10). Experiences from the United Kingdom show how electronic pathways for STI testing can complement self-testing, with laboratory platforms providing high-quality diagnostics and self-tests delivering immediacy and privacy (28). Comparative research is needed to determine the optimal role of SST within integrated sexual health systems.

Financing and system integration will determine sustainability. Treponemal SSTs must be positioned as entry points within diagnostic pathways, with confirmatory testing embedded in national algorithms (9, 19). To ensure equitable access, national planning should include pooled procurement, subsidization for high-risk groups, and integration into health coverage. By embedding SST into existing procurement and delivery systems, countries can move beyond pilot projects and realize its potential as a scalable public health intervention.

## BROADER IMPLICATIONS AND POLICY RECOMMENDATIONS

The global uptake of COVID-19 self-testing reshaped public expectations for diagnostics, demonstrating that laypersons can reliably perform technically demanding tests when supported with clear instructions, user-friendly design, and accessible follow-up (29, 30). This widespread experience with at-home testing created a precedent that can be leveraged to normalize SST in contexts where conventional models have struggled to meet rising demand.

Importantly, SSTs should not be considered stand-alone diagnostics. As treponemal assays cannot distinguish between past and current infection, all reactive results require confirmatory testing within national algorithms, typically involving both treponemal and lipoidal assays in clinical settings (9, 19). SST should, therefore, be framed as an entry point into the diagnostic cascade, facilitating earlier engagement with care and reducing delays, rather than as a replacement for facility-based diagnosis.

Policy priorities must embed SST within systems that guarantee equity, quality, and sustainability. Integration of SST results into national surveillance platforms is needed for epidemic monitoring. Distribution should extend beyond pharmacies and online retail to include public-sector outlets, community-based organizations, and peer-led channels, with safeguards to protect voluntariness. National procurement strategies can leverage pooled purchasing with HIV diagnostics to reduce costs, while public financing or insurance coverage is needed to avoid inequities from out-of-pocket purchase. Policy frameworks should also mandate accessible design features such as intuitive visuals and simplified instructions to support persons with disabilities, older adults, and marginalized groups (22). Finally, operational research should evaluate bundled HIVST–SST platforms, digital navigation tools, and community-led models. These efforts can generate evidence on scalable pathways that balance access, cost, and quality across diverse health systems.

## CONCLUSION

The FDA approval of the first SST provides new momentum for decentralized STI service delivery. Similar approaches could be applied across diverse contexts. Realizing the full public health potential of SST, however, will require addressing key implementation challenges: ensuring strong linkage to care, supporting vulnerable users, sustaining financing, and embedding quality assurance at every level. The lessons of HIVST remind us that innovation alone is not enough; success depends on ongoing engagement, adaptation, and a commitment to equity. As we stand at the threshold of a new era in syphilis control, stakeholders must act with urgency and care to ensure that self-testing becomes a tool for empowerment, prevention, and health equity for all.
